# Virulence Genotyping of *Pasteurella multocida* Isolated from Multiple Hosts from India

**DOI:** 10.1155/2014/814109

**Published:** 2014-11-18

**Authors:** Laxmi Narayan Sarangi, Adyasha Priyadarshini, Santosh Kumar, Prasad Thomas, Santosh Kumar Gupta, Viswas Konasagara Nagaleekar, Vijendra Pal Singh

**Affiliations:** ^1^Division of Veterinary Bacteriology and Mycology, Indian Veterinary Research Institute, Izatnagar, Bareilly, Uttar Pradesh 243122, India; ^2^Regional Medical Research Centre, Bhubaneswar, Odisha 751023, India; ^3^Institute of Bacterial Infections and Zoonoses, Friedrich Loeffler Institut, Namburger Straße 96 a, 07743 Jena, Germany

## Abstract

In this study, 108 *P. multocida* isolates recovered from various host animals such as cattle, buffalo, swine, poultry (chicken, duck, and emu) and rabbits were screened for carriage of 8 virulence associated genes. The results revealed some unique information on the prevalence of virulence associated genes among Indian isolates. With the exception of *toxA* gene, all other virulence associated genes were found to be regularly distributed among host species. Association study between capsule type and virulence genes suggested that *pfhA, nanB,* and *nanH* genes were regularly distributed among all serotypes with the exception of CapD, whereas *toxA* gene was found to be positively associated with CapD and CapA. The frequency of *hgbA* and *nanH* genes among swine isolates of Indian origin was found to be less in comparison to its equivalents around the globe. Interestingly, very high prevalence of *tbpA* gene was observed among poultry, swine, and rabbit isolates. Likewise, very high prevalence of *pfhA* gene (95.3%) was observed among Indian isolates, irrespective of host species origin.

## 1. Introduction

*Pasteurella multocida* belonging to family Pasteurellaceae is a ubiquitous organism affecting multihost species, thus causing several diseases like haemorrhagic septicaemia in cattle and buffalo, enzootic bronchopneumonia in cattle, sheep, and goats, atrophic rhinitis in swine, fowl cholera in poultry and snuffles in rabbits [1, 2]. These diseases are known to cause severe financial loss to livestock industry, especially in tropical countries. Conventional vaccines have been used for several decades as a control strategy, but major limitation of these vaccines is their ineffectiveness in inducing long acting cross protective immunity [3, 4]. Therefore, several outer membrane proteins (OMPs) have been proposed as candidate antigen for subunit vaccine [3, 5].

The OMPs of Gram negative bacteria play an essential role in the disease process. They are involved in the process of nutrient uptake, transport of molecules in and out of the cell, colonization and invasion of the host, evasion of host immune response, injury to host tissue, and so forth, required for productive infection [6]. These proteins are subjected to different selection pressures, thereby exhibiting varying degree of interstrain heterogeneity. Therefore, these virulence associated genes can be used to assess intraspecies diversity and also to obtain epidemiological relationships [7]. In addition, these OMPs are good immunogens and can be used as vaccine components to provide protection [8–10]. Hence, virulence profiling can be used as a typing method for characterization of bacterial pathogens [11] and also for development of subunit vaccine in vaccine strain selection. For the first time, virulence profiling of* P. multocida* isolates was carried out by Ewers et al. [12], and subsequently it has been used by many workers to understand the diversity of the pathogen recovered from different host origin [13–19].

The previous study carried out in our laboratory on carriage of 19 virulence genes among* P. multocida* isolates, recovered from small ruminants, revealed some novel information on the frequency of virulence genes like very high prevalence of* pfhA* gene, 48.9% prevalence of* toxA* gene with the highest prevalence among serotype A followed by serotype D, and in one isolate each of capsular types B and F (Sarangi et al., submitted for publication). These findings from small ruminant isolates encouraged sampling of more isolates of Indian origin from various hosts to have a clear understanding on the heterogeneity of the bacteria. Therefore, this study was extended to* P. multocida* isolates recovered from multiple host species for 8 important virulence associated genes, encoding proteins involved in bacterial survival and pathogenesis. It included genes encoding transferrin binding protein (TbpA) and haemoglobin binding protein (HgbA, HgbB) associated with iron acquisition, filamentous haemagglutinin (PfhA), subunit of type IV fimbriae (PtfA), sialidases (NanB, NanH) involved in initial colonization and adhesion, and dermonecrotoxin (ToxA).

## 2. Materials and Methods

### 2.1. Bacterial Strains

In the present study, 108* P. multocida* isolates recovered from large ruminants (buffalo, *n* = 23, cattle, *n* = 18), avians (chicken, *n* = 18, duck, *n* = 8, and emu, *n* = 4), swine (*n* = 34), and rabbit (*n* = 3), maintained at Division of Bacteriology & Mycology, Indian Veterinary Research Institute, Izatnagar, were used. Selections of isolates were carried out on the basis of host origin, year and place of isolation in order to incorporate isolates from all over India. The details of the isolates (isolate number, host origin, capsular type, year and place of isolation, and disease symptom (if available)) are given in Table 1.

### 2.2. Confirmation of* P. multocida* Isolates

The isolates were revived in brain heart infusion broth by 18–24 h incubation at 37°C and plated subsequently onto blood agar to study cultural characteristics. The cultures were then tested for purity by biochemical tests as per standard techniques [20]. The genomic DNA of the isolates was extracted by CTAB method [21], and the isolates were reconfirmed as* P. multocida* by PM-PCR followed by determination of capsular type by multiplex PCR [22, 23].

### 2.3. Detection of Virulence Associated Genes by PCR

The isolates were then subjected to screening of 8 virulence genes encoding iron binding proteins (TbpA, HgbA, HgbB), colonization and adhesion related protein (PfhA, PtfA), sialidases (NanB, NanH), and dermonecrotoxin (ToxA) by individual PCR reactions, utilizing oligonucleotide primers described previously. The details of the virulence genes, sequences of the oligonucleotide primers, and citations used are listed in Table 2.

### 2.4. Statistical Analysis

Statistical analysis of the data generated from the study was performed with SPSS 16.0 (SPSS Inc., Chicago). *P* values of <0.05 were considered as statistically significant.

## 3. Results and Discussion

*P. multocida* is an economically important veterinary pathogen, causing wide range of diseases in livestock and poultry. The bacteria have been classified into five capsular types (A, B, D, E, and F) based on capsular typing, with each capsule type being predominantly associated with a particular disease in a host species. But isolation of other capsular types from such hosts by cross species infection is not uncommon [2, 7]. Ability of the bacteria to infect and survive in several hosts as commensal exposes it to various selection pressures, resulting in emergence of divergent strains in field scenario. Molecular epidemiological study by employing REP-PCR, ERIC-PCR, MLST analysis, and so forth has confirmed the diversity of* P. multocida* circulating in India and also the possibility of transboundary spread of strains across evolutionary time [24, 25]. Therefore, a detailed study on the presence of virulence associated genes recovered from different host species in Indian subcontinent will be helpful to understand the disease process and to develop disease control measures in future.

In this study,* P. multocida* isolates recovered from various host species were screened for presence of 8 important virulence associated genes (*tbpA, hgbA, hgbB, pfhA, ptfA, nanB, nanH,* and* toxA*) involved in bacterial pathogenesis. The results confirmed that, with the exception of* toxA* gene, all other virulence associated genes are regularly distributed among the isolates of different host origin. The result of individual PCR reaction for each isolate is presented in Table 1. Among the genes encoding iron binding proteins,* tbpA* gene was present in 82.4% of isolates which range from 69.6% in buffalo to 100% in cattle, emu, and rabbits (Table 3). Similarly,* hgbA* gene was found to be regularly distributed among all isolates affecting different hosts with the lowest prevalence among swine isolates (73.5%). Gene* hgbB* has the lowest prevalence among the three iron binding proteins screened in this study and was found in 72.2% of the isolates. The percentage prevalence of this gene was found to be more among avian isolates (90%) in comparison to large ruminants (65.9%) and swine (67.6%) isolates. Of the two sialidases present in* P. multocida* isolates, the percentage prevalence of* nanB* gene was found to be more than* nanH* gene. Overall very high prevalence of* pfhA* gene (93.5%) was observed in this study with 100% prevalence among avian isolates. Dermonecrotoxin gene (*toxA*) was found only in 17.6% of strains with majority of the isolates belonging to porcine origin. One buffalo and 3 duck isolates were also found to carry* toxA* gene (Table 3).

Iron acquisition and uptake are essential for bacterial survival, and many bacteria have developed different iron sequestering system for uptake of iron. The expression of iron acquisition proteins increases under iron limiting condition, as well as* in vivo* condition (reviewed in [5]).* P. multocida* utilizes various receptors for adapting to variations in supply of different haem iron sources [5]. Among these, TbpA protein is necessary for extraction of iron from transferrin and has been reported to be an important virulence factor and epidemiological marker in cattle [12, 19, 26]. Previous studies reported that* tbpA* gene is either absent or rarely present in poultry, swine, and rabbit isolates (Table 4) [12–16]. In contrast to these findings, we observed a very high occurrence of* tbpA* gene among poultry (83.3%), swine (79.4%), and rabbit (100%) isolates (Tables 3 and 4). Among the host species, the prevalence of this gene was found to be lowest (69.6%) in buffalo (Table 3). The difference in prevalence of this gene among isolates of cattle and buffalo origin was found to be statistically significant (*P* < 0.05), which is quite unexpected. Therefore more number of isolates from both host origins should be carried out before reaching any conclusion. In this study,* tbpA* gene was found to be frequently distributed among four capsule types, including CapF (Table 3). This is in contrast to Ewers et al. [12], who observed* tbpA* gene in 70% of CapB strains, followed by 37% of CapA, 9.5% of CapD strains and nil in CapF strains. Similarly, Katsuda et al. [18] reported positive association of CapA strain with* tbpA* gene.

*P. multocida* utilizes two proteins (HgbA and HgbB) for acquiring iron directly from haem component. Morton et al. [27] reported that the presence of both proteins might provide increased uptake of iron and protection against negative effects of mutation in one of the encoding genes. Between these two proteins,* hgbA* gene was found to be regularly distributed (>95% prevalence) among isolates [12–15, 17, 18]. In the present study, 73.5% of porcine isolates were found to carry this gene, which is lower in comparison to previous findings, that is, nearly 100% prevalence (Table 4) [12–15]. The frequency of* hgbB* gene varies among strains of different host origin and also with disease status of the animal [12, 15, 16, 18, 19]. In this study, 72.2% of the isolates were found to carry this gene with highest frequency observed among avian strains (90%), which is in agreement with previous reports (Table 4) [12, 17].

Among the genes encoding proteins involved in bacterial colonization and adhesion,* ptfA* gene has the highest (98.1%) prevalence (Table 3). This gene encodes type 4 fimbria subunit and has been associated with bovine diseases [19]. Worldwide, this gene is regularly distributed with more than 85% prevalence among* P. multocida* isolates, irrespective of host origin and capsule type (Table 4).

*pfhA *gene encoding filamentous haemagglutinin is an important epidemiological marker and the presence of this gene has been correlated with occurrence of disease in cattle, swine and sheep [12, 13, 18, 19, 28]. Almost all previous studies reported low prevalence of this gene with varying frequencies in between 46–52%, 45–60%, and 15–40.5% among isolates of cattle, poultry, and pig origin, respectively (Table 4) [12–15]. But interestingly, very high prevalence, 85.3% (pig) to 100% (avian), of this gene was observed among Indian isolates (Table 3). This suggests* pfhA* gene might be providing survival advantage to the bacterium in the host and the occurrence of horizontal gene transfer has led to such high prevalence among Indian strains/clones.

Sialidases play an important role in colonization to epithelial surface. They enhance bacterial virulence by unmasking key host receptor and by reducing the effectiveness of mucin [5, 29]. Of the two sialidases (NanB and NanH) present in* P. multocida* isolates, the* nanB* gene was found in almost all isolates, whereas the prevalence of* nanH* varied according to host origin and geographical location. In this study, the frequency of* nanH* gene among poultry isolates was found to be low (63.3%), which is in contrast to the report of Furian et al. [17] (Table 4). Similarly, the carriage of* nanH* gene among isolates of pig origin from India was also found to be lower (67.6%) in comparison to isolates from other parts of the globe, which reported higher (>97%) frequency (Table 4) [12–15].

Dermonecrotoxin (sometimes called* P. multocida* toxin) is encoded by* toxA* gene. This gene was initially detected in serotype D isolates and was found to be associated with atrophic rhinitis in pigs. Later on, it was detected in strains of serotype A from pigs and other hosts [30]. In this study,* toxA* gene was detected in 44.1% of pig isolates. Further, one buffalo and three duck isolates were also found positive for* toxA* gene (Table 3). Two serotype B isolates were found to carry this gene which is in agreement with our previous findings (Sarangi et al., submitted for publication). A lysogenic bacteriophage infection of* P. multocida* resulting in horizontal gene transfer could be the reason [31].

The association of virulence associated genes with particular capsular type and host origin was assessed by the Chi-square and Fisher's exact test. Out of the 8 virulence associated genes studied* toxA, pfhA, nanB,* and* nanH* were found to be associated (positive or negative) with capsular type.* pfhA, nanB,* and* nanH* genes were found to be regularly distributed among all serotypes with the exception of serotype D. Negative association of* pfhA* gene with CapD strains has been reported previously [12, 14, 18]. Dermonecrotoxin encoded by* toxA *gene was found to be positively associated with capD and CapA. Ewers et al. [12] observed clear association of* toxA* gene with CapD strains which was later supported by similar reports from other workers [13, 14]. Among cattle isolates, a significant difference (*P* = 0.021) was observed in the distribution of* hgbB* gene among serotypes (Table 3). Similarly, for pig isolates the frequency of* pfhA* and* nanH* gene among serotypes was found to be statistically significant (Table 3). However, as the number of strains tested under each serotype was less, more number of samples should be tested before reaching any definite conclusion. In order to ascertain any trend in the distribution of virulence genes over time period, the strains used in the study were divided into two groups, contemporary (2009–2014) and archived (1992–2008), and statistical analysis was carried out. But no statistically significant difference was observed between the two groups with respect to virulence gene distribution (Table 1).

The prevalence of virulence associated genes was found to vary among* P. multocida* isolates recovered from various host species. Significant association between* toxA* and* nanH* genes with host origin was also observed. Dermonecrotoxin gene was found to be positively associated with porcine isolates, whereas* nanH* gene was found to be positively associated with large ruminant isolates, more specifically with cattle isolates, which agrees well with the findings of Ewers et al. [12].

The combination of genes among* P. multocida* isolates was assessed by the Chi-square and Fisher's exact test. Significant association was observed between* tbpA-hgbA, tbpA-hgbB, tbpA-pfhA, tbpA-nanB, tbpA-nanH, hgbA-hgbB, hgbA-pfhA, hgbA-nanB, hgbA-nanH, pfhA-nanB,* and* pfhA-nanH*. Similar association among iron acquisition genes, as well as between various virulence associated genes, has been reported previously by Ewers et al. [12].

To sum up, the present study revealed some unique epidemiological information on the prevalence of virulence associated genes among Indian strains in comparison to its equivalents in other parts of the globe. The result shows that with the exception of* toxA* gene the virulence associated genes are regularly distributed among* P. multocida* isolates. The occurrence of* ptfA, hgbA,* and* nanH* genes among swine isolates of Indian origin was found to be less in comparison to other countries. Gene encoding dermonecrotoxin was observed in 17.6% of the total isolates studied. This gene is present mostly among swine isolates, with few occurrences in buffalo and duck isolates. Interestingly, very high prevalence of* tbpA* gene was observed among poultry, swine, and rabbit isolates. Likewise, very high prevalence of* pfhA* gene was observed among Indian isolates, irrespective of host species origin. As proper history of majority of the isolates with respect to its disease status was not available, it was not possible to perform association study between virulence gene and disease status of the animal, which could have enhanced the significance of this study. Therefore, more number of isolates with proper history on disease status of the host should be carried out in future, which will be helpful to make a more definite conclusion, to provide insight into mechanism of pathogenesis, association of genes with outcome of the disease, and in future vaccine strategies.

## Figures and Tables

**Table 1:** Details of the isolates (host origin, serotype detected, place of isolation, year of isolation, symptom, presence/absence of individual virulence genes).

Sample id	Species	Serotype	Place	Year	Disease/symptom	*tbpA *	*hgbA *	*hgbB *	*pfhA *	*ptfA *	*toxA *	*nanB *	*nanH *
10	Cattle	F	Pune	1992	N.A.	P	P	P	P	P	A	P	P
11	Cattle	F	Pune	1992	N.A.	P	P	P	P	P	A	P	P
51	Pig	A	UP	1995	N.A.	P	P	P	P	P	P	P	A
53	Buffalo	B	Pune	1996	N.A.	A	P	P	P	P	A	P	A
98	Duck	A	Tripura	2001	N.A.	P	P	P	P	P	A	P	P
117	Cattle	B	Bhubaneswar	2001	N.A.	P	P	P	P	P	A	P	P
118	Cattle	B	Bhubaneswar	2001	N.A.	P	P	P	P	P	A	P	P
120	Cattle	B	Bhubaneswar	2001	N.A.	P	P	P	P	P	A	P	P
128	Cattle	B	Bangalore	2001	N.A.	P	P	P	P	P	A	P	P
132	Buffalo	A	Palampur	2001	N.A.	P	P	P	P	P	A	P	P
133	Buffalo	A	Palampur	2001	N.A.	A	A	P	P	P	A	P	A
134	Buffalo	A	Palampur	2001	N.A.	P	P	P	P	P	A	P	P
141	Chicken	A	Chennai	2001	N.A.	P	P	P	P	P	A	P	P
202	Chicken	A	Chennai	2002	N.A.	A	P	P	P	P	A	P	A
206	Chicken	B	Chennai	2002	N.A.	P	P	P	P	P	A	P	A
222	Buffalo	A	Mathura	2002	N.A.	P	P	A	P	P	A	P	P
258	Chicken	A	Nasik	2002	N.A.	P	P	P	P	P	A	P	P
330	Chicken	B	Anand	2002	N.A.	P	P	P	P	P	A	P	P
288	Buffalo	B	Bhubaneswar	2003	N.A.	P	P	P	P	P	A	P	P
291	Pig	B	Guwahati	2003	N.A.	P	P	A	P	P	A	P	P
292	Pig	B	Guwahati	2003	N.A.	P	P	P	P	P	A	P	A
366	Cattle	B	Palampur	2004	N.A.	P	A	A	P	P	A	P	P
390	Buffalo	B	Palampur	2005	N.A.	P	P	P	P	P	A	P	P
400	Buffalo	B	Ludhiana	2005	N.A.	P	P	P	P	P	A	P	P
407	Chicken	B	Ludhiana	2005	N.A.	P	P	P	P	P	A	P	P
409	Buffalo	B	Jammu	2005	N.A.	P	P	P	P	P	A	P	P
410	Buffalo	B	Jammu	2005	N.A.	P	P	P	P	P	A	P	P
425	Duck	B	Chennai	2005	N.A.	P	P	P	P	P	P	P	P
448	Rabbit	B	Palampur	2006	N.A.	P	P	A	P	P	A	P	P
456	Chicken	A	Chennai	2006	N.A.	P	P	P	P	P	A	P	P
460	Chicken	A	Chennai	2006	N.A.	A	A	A	P	P	A	P	A
464	Chicken	A	Chennai	2006	N.A.	A	P	P	P	P	A	P	P
569	Chicken	A	Chennai	2007	N.A.	P	A	A	P	P	A	P	A
537	Pig	A	Guwahati	2007	N.A.	A	A	A	P	P	A	A	P
540	Pig	A	Guwahati	2007	N.A.	P	P	P	P	P	P	P	P
543	Pig	D	Guwahati	2007	N.A.	P	P	P	P	P	P	P	A
550	Duck	A	Guwahati	2007	N.A.	P	A	P	P	P	P	P	P
555	Buffalo	B	Anand	2007	N.A.	P	P	A	P	P	A	P	A
559	Rabbit	B	Palampur	2007	Nasal discharge	P	P	A	P	P	A	P	P
563	Cattle	B	Anand	2007	N.A.	P	P	A	P	P	A	P	P
585	Pig	A	Guwahati	2008	N.A.	P	P	A	P	P	P	P	P
587	Pig	A	Guwahati	2008	N.A.	P	P	P	P	P	P	P	P
602	Buffalo	B	Palampur	2008	N.A.	P	P	P	P	P	A	P	P
608	Rabbit	A	Palampur	2008	Nasal discharge	P	P	P	P	P	A	P	P
610	Buffalo	B	Ludhiana	2008	N.A.	A	A	A	P	P	A	P	A
618	Chicken	B	Palampur	2008	N.A.	P	P	P	P	P	A	P	P
632	Buffalo	B	Anand	2008	N.A.	A	A	A	P	P	A	P	P
633	Chicken	B	Bangalore	2008	N.A.	P	A	P	P	P	A	P	P
655	Buffalo	D	Guwahati	2008	N.A.	P	P	P	P	P	A	P	P
701	Pig	A	Guwahati	2009	N.A.	P	P	A	P	P	P	P	P
702	Pig	A	Guwahati	2009	N.A.	P	P	P	P	P	A	P	P
703	Pig	D	Guwahati	2009	N.A.	P	P	P	P	P	P	P	A
704	Cattle	B	Guwahati	2009	N.A.	P	P	A	A	P	A	P	P
720	Pig	B	UP	2009	N.A.	P	P	P	P	P	A	P	P
721	Pig	B	UP	2009	N.A.	P	P	P	P	P	A	P	P
722	Pig	B	UP	2009	N.A.	P	P	A	P	P	P	P	P
725	Buffalo	A	Ludhiana	2009	N.A.	P	P	P	P	P	P	P	A
733	Pig	D	Guwahati	2009	N.A.	P	A	P	P	P	P	P	A
736	Pig	D	Guwahati	2009	N.A.	P	P	P	A	P	A	P	A
737	Pig	D	Guwahati	2009	N.A.	P	P	P	A	P	A	P	A
749	Cattle	A	Palampur	2009	N.A.	P	P	P	P	P	A	P	P
746	Cattle	A	Palampur	2009	N.A.	P	P	P	P	P	A	P	P
747	Cattle	A	Palampur	2009	N.A.	P	P	P	P	A	A	P	P
754	Cattle	A	Palampur	2009	N.A.	P	P	P	P	P	A	P	P
782	Chicken	A	Anand	2009	N.A.	A	P	A	P	P	A	P	A
794	Chicken	A	Thrissur	2009	Necrotic foci in liver and haemorrhage in heart	P	P	P	P	P	A	P	A
652	Buffalo	B	Guwahati	2010	N.A.	P	P	P	P	P	A	P	P
653	Buffalo	B	Guwahati	2010	N.A.	P	P	P	P	P	A	P	P
784	Chicken	A	Anand	2010	N.A.	P	P	P	P	P	A	P	P
803	Chicken	A	Chennai	2010	N.A.	P	P	P	P	P	A	P	P
804	Chicken	A	Anand	2010	N.A.	P	P	P	P	P	A	P	P
811	Cattle	A	Palampur	2010	N.A.	P	P	P	P	P	A	P	P
852	Pig	A	Guwahati	2011	Diseased	P	P	P	P	P	P	P	P
860	Pig	B	Guwahati	2011	Diseased	P	P	P	P	P	A	P	P
876	Pig	A	Thrissur	2011	Fever	A	A	A	P	P	A	A	P
877	Pig	A	Thrissur	2011	Fever	A	A	P	P	P	A	P	P
879	Pig	A	Thrissur	2011	Fever	A	P	A	P	P	A	P	A
890	Emu	A	Chennai	2011	N.A.	P	P	P	P	P	A	P	P
2751	Cattle	B	Palampur	2011	Nasal discharge	P	P	A	P	P	A	P	P
2766	Cattle	B	Palampur	2011	Nasal discharge	P	P	P	P	P	A	P	P
3324	Cattle	B	Palampur	2011	Nasal discharge	P	P	A	P	P	A	P	P
4312	Cattle	B	Palampur	2011	Nasal discharge	P	P	A	P	P	A	P	P
BP23	Pig	B	Guwahati	2011	N.A.	P	A	A	P	P	A	P	P
BP28	Pig	A	Guwahati	2011	N.A.	P	A	P	P	P	P	P	P
BP37	Pig	A	Guwahati	2011	N.A.	P	P	A	P	P	P	P	P
EMU 2	Emu	A	Chennai	2011	N.A.	P	P	P	P	P	A	P	P
JP18	Pig	A	Guwahati	2011	N.A.	P	P	P	P	P	P	P	P
NP23	Pig	B	Guwahati	2011	N.A.	P	P	P	P	P	A	P	P
NP37	Pig	B	Guwahati	2011	N.A.	P	P	P	P	P	A	P	P
PP1A	Pig	A	Thrissur	2011	N.A.	P	P	P	P	P	A	P	P
PP2A	Pig	A	Thrissur	2011	N.A.	P	P	P	P	P	P	P	P
PP4A	Pig	A	Thrissur	2011	N.A.	P	P	P	P	P	A	P	P
914	Duck	A	Thrissur	2012	N.A.	P	P	P	P	P	A	P	P
920	Emu	A	Anand	2012	N.A.	P	P	P	P	P	A	P	P
922	Emu	A	Anand	2012	N.A.	P	P	P	P	P	A	P	P
DP53	Duck	A	Thrissur	2013	N.A.	P	P	P	P	P	A	P	A
DP54	Duck	A	Thrissur	2013	N.A.	P	P	P	P	P	P	P	A
DP55	Duck	A	Thrissur	2013	N.A.	A	P	P	P	P	A	P	A
DP56	Duck	A	Thrissur	2013	N.A.	P	P	P	P	P	A	P	A
P14	Pig	D	Guwahati	2013	N.A.	A	A	A	A	P	P	A	A
P15	Pig	A	Guwahati	2013	N.A.	A	A	A	A	P	A	P	A
P16	Pig	D	Guwahati	2013	N.A.	A	A	P	A	P	A	A	A
PAB 78	Buffalo	B	Anand	2013	N.A.	P	P	A	P	P	A	P	P
PAB 80	Buffalo	B	Anand	2013	N.A.	P	P	A	P	P	A	P	P
PAB 86	Buffalo	B	Anand	2013	N.A.	A	A	A	A	A	A	P	P
PAP 88	Chicken	A	Anand	2013	N.A.	P	P	P	P	P	A	P	A
LDHB 106	Buffalo	B	Ludhiana	2014	N.A.	A	P	A	P	P	A	P	P
MSRB 108	Buffalo	B	Ludhiana	2014	N.A.	A	P	P	P	P	A	P	P

(N.A. = not available; A = absence of virulence gene; P = presence of virulence gene as detected in PCR reaction).

**Table 2:** Details of primers and citations used for the detection of capsular type and virulence associated genes in strains of *Pasteurella multocida*.

Gene	Primer	Primer sequence (5′-3′)	Reference
PM-PCR and Capsular serotypes			
*KMT1 *	PMPCR-F PMPCR-R	ATCCGCTATTTACCCAGTGG GCTGTAAACGAACTCGCCAC	[22]
*hyaD-hyaC *	capA F capA R	GATGCCAAAATCGCAGTCAG TGTTGCCATCATTGTCAGTG	[23]
*bcbD *	capB F capB R	CATTTATCCAAGCTCCACC GCCCGAGAGTTTCAATCC	[23]
*dcbF *	capD F capD R	TTACAAAAGAAAGACTAGGAGCCC CATCTACCCACTCAACCATATCAG	[23]
*ecbJ *	capE F capE R	TCCGCAGAAAATTATTGACTC GCTTGCTGCTTGATTTTGTC	[23]
*fcbD *	capF F capF R	AATCGGAGAACGCAGAAATCAG TTCCGCCGTCAATTACTCTG	[23]

Iron acquisition genes			
*tbpA *	tbpA F tbpA R	GGACAGTGCATATAACTTGTT GGACAGTGCATATAACTTGTTTACTA	[32]
*hgbA *	hgbA F hgbA R	CATATCGGATCCTTGAAACCAGAGGAAGCAAAAA GAATCGGAGCTCACGACCTGGTGAGTAAAAACGAT	In-house [33]
*hgbB *	HgbB F HgbB R	ACCGCGTTGGAATTATGATTG CATTGAGTACGGCTTGACAT	[12]

Adhesins			
*ptfA *	ptfA F ptfA R	AGGATCCATGAAAAAAGCCATTT GGAGCTCTTATGCGCAAAATCCTG	In-house
*pfhA *	pfhA F pfhA R	TAAGCCTATCGGTTCAAGTCG GATAAATCTACCCCGTCCTCT	In-house

Sialidases			
*NanB *	NanB F NanB R	GTCCTATAAAGTGACGCCGA ACAGCAAAGGAAGACTGTCC	[12]
*nanH *	nanH F nanH R	CACTGCCTTATAGCCGTATTC AGCACTGTTACCCGAACCC	[12]

Dermonecrotoxin			
*ToxA *	ToxA F ToxA R	TCTTAGATGAGCGACAAGG GAATGCCACACCTCTATAG	[34]

**Table 3:** Prevalence of virulence associated genes among *Pasteurella multocida* isolates recovered from various host species of India.

Host origin/capsular type	No. of strains	*tbpA *(%)	*hgbA *(%)	*hgbB* (%)	*pfhA* (%)	*ptfA* (%)	*nanB* (%)	*nanH* (%)	*toxA* (%)

**Buffalo**	**23**	**69.6**	**82.6**	**65.2**	**95.7**	**95.7**	**100**	**78.3**	**4.3**
Cap type A	5	80.0	80.0	80.0	100	100	100	60.0	20.0
Cap type B	17	64.7	82.3	58.8	94.1	94.1	100	82.3	0.0
Cap type D	1	100	100	100	100	100	100	100	0.0
Cap type F	0	N.A.	N.A.	N.A.	N.A.	N.A.	N.A.	N.A.	N.A.

**Cattle**	**18**	**100**	**94.4**	**66.7**	**94.4**	**94.4**	**100**	**100**	**0.0**
Cap type A	5	100	100	**100**	100	80.0	100	100	0.0
Cap type B	11	100	90.9	**45.4**	90.9	100	100	100	0.0
Cap type D	0	N.A.	N.A.	**N.A.**	N.A.	N.A.	N.A.	N.A.	N.A.
Cap type F	2	100	100	**100**	100	100	100	100	0.0

**Large ruminants (cattle + buffalo)**	**41**	**82.9**	**87.8**	**65.9**	**95.1**	**95.1**	**100**	**87.8**	**2.4**
Cap type A	10	90.0	90.0	90.0	100	90.0	100	80.0	10.0
Cap type B	28	78.5	85.7	53.5	92.8	96.4	100	89.2	0.0
Cap type D	1	100	100	100	100	100	100	100	0.0
Cap type F	2	100	100	100	100	100	100	100	0.0

**Chicken**	**18**	**77.8**	**83.3**	**83.3**	**100**	**100**	**100**	**61.1**	**0.0**
Cap type A	13	69.2	84.6	76.9	100	100	100	53.8	0.0
Cap type B	5	100	80.0	100	100	100	100	80.0	0.0
Cap type D	0	N.A.	N.A.	N.A.	N.A.	N.A.	N.A.	N.A.	N.A.
Cap type F	0	N.A.	N.A.	N.A.	N.A.	N.A.	N.A.	N.A.	N.A.

**Duck**	**8**	**87.5**	**87.8**	**100**	**100**	**100**	**100**	**50.0**	**37.5**
Cap type A	7	85.7	85.7	100	100	100	100	42.8	28.5
Cap type B	1	100	100	100	100	100	100	100	100
Cap type D	0	N.A.	N.A.	N.A.	N.A.	N.A.	N.A.	N.A.	N.A.
Cap type F	0	N.A.	N.A.	N.A.	N.A.	N.A.	N.A.	N.A.	N.A.

**Emu**	**4**	**100**	**100**	**100**	**100**	**100**	**100**	**100**	**0.0**
Cap type A	4	100	100	100	100	100	100	100	0.0
Cap type B	0	N.A.	N.A.	N.A.	N.A.	N.A.	N.A.	N.A.	N.A.
Cap type D	0	N.A.	N.A.	N.A.	N.A.	N.A.	N.A.	N.A.	N.A.
Cap type F	0	N.A.	N.A.	N.A.	N.A.	N.A.	N.A.	N.A.	N.A.

**Avian (chicken + duck + emu)**	**30**	**83.3**	**86.7**	**90.0**	**100**	**100**	**100**	**63.3**	**10.0**
Cap type A	24	79.1	87.5	87.5	100	100	100	58.3	8.3
Cap type B	6	100	83.3	100	100	100	100	83.3	16.6
Cap type D	0	N.A.	N.A.	N.A.	N.A.	N.A.	N.A.	N.A.	N.A.
Cap type F	0	N.A.	N.A.	N.A.	N.A.	N.A.	N.A.	N.A.	N.A.

**Rabbit**	**3**	**100**	**100**	**33.3**	**100**	**100**	**100**	**100**	**0.0**
Cap type A	1	100	100	100	100	100	100	100	0.0
Cap type B	2	100	100	0.0	100	100	100	100	0.0
Cap type D	0	N.A.	N.A.	N.A.	N.A.	N.A.	N.A.	N.A.	N.A.
Cap type F	0	N.A.	N.A.	N.A.	N.A.	N.A.	N.A.	N.A.	N.A.

**Pig**	**34**	**79.4**	**73.5**	**67.6**	**85.3**	**100**	**88.2**	**67.6**	**44.1**
Cap type A	18	72.2	72.2	61.1	**94.4**	100	88.8	**83.3**	44.2
Cap type B	9	100	88.8	66.6	**100**	100	100	**88.8**	11.1
Cap type D	7	71.4	57.1	85.7	**42.8**	100	71.4	**0.0**	57.1
Cap type F	0	N.A.	N.A.	N.A.	**N.A.**	N.A.	N.A.	**N.A.**	N.A.

**Total (all isolates)**	**108**	**82.4**	**83.3**	**72.2**	**93.5**	**98.1**	**96.3**	**75.0**	**17.6**
Cap type A	53	79.2	83.0	79.2	98.0	98.0	96.2	71.7	24.5
Cap type B	45	86.7	86.7	60.0	95.6	97.8	100	88.9	4.4
Cap type D	8	75.0	62.5	87.5	50.0	100	75.0	12.5	50.0
Cap type F	2	100	100	100	100	100	100	100	0.0

**Table 4:** Comparison of the distribution of virulence associated genes among *Pasteurella multocida* isolates recovered from various host species across the globe.

Gene/host	Cattle			Buffalo			Poultry			Pig			Rabbit		
	Country (reference)	No. of strains tested	Prevalence (%)	Country (reference)	No. of strains tested	Prevalence (%)	Country (reference)	No. of strains tested	Prevalence (%)	Country (reference)	No. of strains tested	Prevalence (%)	Country (reference)	No. of strains tested	Prevalence (%)
*tbpA *	India (this study)	18	100	India (this study)	23	69.6	India (this study)	30	83.3	India (this study)	34	79.4	India (this study)	03	100
	Germany [12]	104	70.2	Germany [12]	07	57.1	Germany [12]	20	0.0	Germany [12]	52	0.0	Germany [12]	20	0.0
	Japan [18]	378	76.2							Spain [15]	205	0.0	Brazil [16]	46	8.6
	India [19]	23	100							Germany [13]	382	0.0			

*hgbA *	India (this study)	18	94.4	India (this study)	23	82.6	India (this study)	30	86.7	India (this study)	34	73.5	India (this study)	03	100
	Germany [12]	104	95.2	Germany [12]	07	100	Germany [12]	20	90.0	Germany [12]	52	98.1	Germany [12]	20	100
	Japan [18]	378	95.5				Brazil [17]	25	100	Spain [15]	205	100	Brazil [16]	46	73.9
	India [19]	23	100							Germany [13]	382	100			
										China [14]	233	96.6			

*hgbB *	India (this study)	18	66.7	India (this study)	23	65.2	India (this study)	30	90.0	India (this study)	34	67.6	India (this study)	03	33.3
	Germany [12]	104	57.7	Germany [12]	07	85.7	Germany [12]	20	85.0	Germany [12]	52	86.5	Germany [12]	20	100
	Japan [18]	378	61.4				Brazil [17]	25	100	Spain [15]	205	60.5	Brazil [16]	46	30.4
	India [19]	23	26.1							Germany [13]	382	84.3			

*ptfA *	India (this study)	18	94.4	India (this study)	23	95.7	India (this study)	30	100	India (this study)	34	100	India (this study)	03	100
	Germany [12]	104	99.0	Germany [12]	07	100	Germany [12]	20	100	Germany [12]	52	100	Germany [12]	20	100
	Japan [18]	378	94.7				Brazil [17]	25	92.0	Spain [15]	205	100	Brazil [16]	46	93.4
	India [19]	23	86.9							Germany [13]	382	100			
										China [14]	233	93.6			

*pfhA *	India (this study)	18	94.4	India (this study)	23	95.7	India (this study)	30	100	India (this study)	34	85.3	India (this study)	03	100
	Germany [12]	104	46.2	Germany [12]	07	100	Germany [12]	20	45.0	Germany [12]	52	21.2	Germany [12]	20	75.0
	Japan [18]	378	52.4				Brazil [17]	25	60.0	Spain [15]	205	40.5	Brazil [16]	46	0.0
	India [19]	23	100							Germany [13]	382	20.9			
										China [14]	233	15.0			

*nanB *	India (this study)	18	100	India (this study)	23	100	India (this study)	30	100	India (this study)	34	88.2	India (this study)	03	100
	Germany [12]	104	100	Germany [12]	07	100	Germany [12]	20	100	Germany [12]	52	100	Germany [12]	20	100
	Japan [18]	378	100				Brazil [17]	25	100	Spain [15]	205	100	Brazil [16]	46	95.6
	India [19]	23	0.0							Germany [13]	382	100			
										China [14]	233	81.5			

*nanH *	India (this study)	18	100	India (this study)	23	78.3	India (this study)	30	63.3	India (this study)	34	67.6	India (this study)	03	100
	Germany [12]	104	88.5	Germany [12]	07	100	Germany [12]	20	65.0	Germany [12]	52	98.1	Germany [12]	20	100
	Japan [18]	378	88.4				Brazil [17]	25	96.0	Spain [15]	205	100	Brazil [16]	46	67.3
	India [19]	23	100							Germany [13]	382	100			
										China [14]	233	97.0			

*toxA *	India (this study)	18	0.0	India (this study)	23	4.3	India (this study)	30	10.0	India (this study)	34	44.1	India (this study)	03	0.0
	Germany [12]	104	5.8	Germany [12]	07	0.0	Germany [12]	20	5.0	Germany [12]	52	36.5	Germany [12]	20	0.0
	India [19]	23	0.0				Brazil [17]	25	0.0	Spain [15]	205	7.8	Brazil [16]	46	0.0
										Germany [13]	382	3.4			
										China [14]	233	4.7			
